# A Case in Practice

**Published:** 1882-09-20

**Authors:** R. L. Hinton

**Affiliations:** Prescott, Ark.


### A CASE IN PRACTICE.

V [Head injury, progressive paralysis.]

By R. L. HINTON, M. D., PRESCOTT, ARK.

July 1st, R. T., an Irishman, æt. 27, living 1½ miles from town, presented himself at my office for treatment—said he had rheumatism; but, having observed his gait, as he approached me, I had already made up my mind that he must be paralyzed in the right leg, which, upon examination, I found to be true, the entire limb being devoid of sensibility and motor-power to about a level with the hip joint. Said he first felt, about a week before, a numbness and tingling in the foot, as "though his foot was asleep," which had gradually extended up. Temperature of the limb, also of the body, normal; pulse, natural; tongue, heavy brown coated. Said he had suffered, in the last few days, considerable pain "in the small of the back." I learned from him, which was afterward corroborated by his friends and relatives who had known him from birth, that he had always been stout and healthy, that he had never had syphilis, scarlatina, or any of those diseases known or supposed to have anything to do with the causation of paralysis. Having seen several cases of local, *temporary* paralysis in connection with our malarial fevers—though I confess I was puzzled to class *this* with these cases—yet, being unable for the time being to trace any other cause, unless it be in some of the nerve centers, I gave him a mercurial for to-night and to-morrow-night, to be followed by quinine each morning, and advised to keep up the quinine, about two six-grain doses every morning, making the doses four hours apart.

July 4. He comes in again; walks much better; feels much better; flatters himself that he is getting well; tongue cleaning off; skin clearer; pulse and body-heat normal; complete insensibility in the entire limb, as before. Same treatment continued.

July 7. Condition about the same, only he feels a tingling and numbness in the left foot, same as in the right foot at first. Add to former treatment

℞ Tr. digitalis,
Fl ext. nux vomica........................aa ℥ j.

Mix. Take six gtt. every six hours, to be increased or decreased, according to symptoms. Counter-irritation to the spine.

He reported to me no more, till on the 16th I was called to see him. I found him flat of his back in bed, both sides paralyzed to about top of the sacrum, involving bowels and bladder; suffering considerably from retention of urine, and some from overloaded bowels. The former promptly relieved by catheter; the latter with some difficulty, after the repetition of cathartics and enemas. There was no sense of feeling, on pricking or pinching the skin, up to the point mentioned on the back; *slight*, on the lower part of abdomen. The tongue slightly coated brown; temperature and pulse natural, after relieving bladder and bowels. Appetite good, and had been about all the time. He now tells me he was taking his medicine, and thought he was getting along very well till, about three days ago, he commenced nursing a very sick relative—was up day and night till he took his bed the day before sending for me. In conversation with the family during this visit, I learned that he received a severe blow on the head about the 2d of April last; that he had not been entirely well since; that the wound was dressed by Drs. Jordan and Sloan, who gave him attention at their office for six weeks. From this date to the time of my first seeing him, about two weeks, he had been at work on the streets, spading, where the sun was very hot, and he constantly stooping, as his work required. On my return to town, I called on Dr. J., who confirmed the statement about the blow on the head; said it was very severe, having cut the scalp to the bone, through a heavy cotton or wool hat, to about three inches in length, just to the right of, and diagonally across the posterior end of the sagital suture; said he was an office patient of his till about the 15th June, during which time he had erysipelas of the scalp, with deep-seated pain in the head, with considerable disturbance of the nervous system, so much so that he had found it necessary to keep him under the influence of the bromides and other quieting remedies all the time, both before and after erysipelas had subsided. As this article is already becoming tedious, I will state that there was nothing very unusual in the symptoms or treatment from this on; digitalis and nux vomica were continued; blisters to the spine, with relieving of the bowels and bladder; also, a trial of electricity, which produced twitching in the muscles of the lower limbs, as did, also, the strychnia. He died at 2 o'clock p. m. on the 20th. I saw him at 9 o'clock a. m. the same day. The paralysis had then reached to a little above the points of the scapula. He was as calm and rational as ever. No change in the general symptoms, only the breathing was a little labored. He remarked to me that the disease was gradually going upward.

On the 21st I was summoned to attend a jury of inquest, and, with the assistance of Drs. Jordan and Sloan, made a *post mortem* examination. On removing the hair, there was the scalp-wound, as already mentioned, now healed. Putrefaction had been very rapid, and the stench was almost intolerable, in spite of disinfectants. Neither plate of the skull was fractured. There was slight adhesion, about the size of a half-dollar, directly under the scalp-wound, of the piamater to the brain; might have been more apparent, had not decay done its work so rapidly.

Here we have a case of progressive paralysis; a man dying by the inch; the work accomplished in twenty days from the first symptom. Will some one or more of our surgeons, who have a more extended field for observation, be kind enough to comment on this case? Tell, at least, whether or not they believe the blow upon the head had anything to do with the disease and death of this man. I, of course, have my views, but it is not best to express them here, as I want the unbiased views of abler ones of the profession. I want it for my own satisfaction and information. The verdict of the jury released the man that struck the blow.

